# Changes in Emotional Demands and Emotion Regulation as Described by Late‐Career Nurses

**DOI:** 10.1002/nop2.70957

**Published:** 2026-10-22

**Authors:** Tímea Zsuzsanna Popucza, Bernice Skytt

**Affiliations:** ^1^ Department of Occupational Health, Psychology and Sport Sciences University of Gävle Gävle Sweden; ^2^ Department of Caring Sciences University of Gävle Gävle Sweden; ^3^ Department of Public Health and Caring Sciences Uppsala University Uppsala Sweden

**Keywords:** ageing, emotion regulation, emotional demands, late‐career nursing, nursing work

## Abstract

**Aim(s):**

This study explored how nurses aged 60 years and older, with at least 20 years of nursing experience, retrospectively described changes in emotional demands and emotion regulation across their careers.

**Design:**

A qualitative interview study using qualitative content analysis.

**Methods:**

Semi‐structured interviews were conducted with 13 active nurses aged 60 years or older in Sweden, each with at least 20 years of nursing experience. The study adhered to the Standards for Reporting Qualitative Research.

**Results:**

Three main categories were identified: professional and personal dimensions, workplace features, and societal transformations. Participants described increasing professional preparedness and more deliberate approaches to emotion regulation over time, alongside changing life circumstances, workplace conditions and societal transformations that shaped emotional demands and opportunities for emotion regulation.

**Conclusion:**

Participants did not attribute changes in emotional demands and emotion regulation solely to chronological age but rather described them in relation to interconnected professional and personal dimensions, workplace features, and broader societal transformations.

**Impact:**

The study challenges simplified age‐based interpretations by showing how late‐career nurses described emotional demands and emotion regulation in relation to changing professional and personal circumstances, workplace conditions and broader societal transformations over the course of long nursing careers. The findings may inform nursing research, leadership and organizational approaches to supporting emotional sustainability.

**Implications for the Profession and/or Patient Care:**

Leadership and organizational practices that acknowledge and address emotional demands within changing healthcare and societal conditions may better support emotional sustainability in nursing. Work design that accommodates changing work roles, recovery needs and opportunities for mentoring may support sustained participation across long nursing careers.

**Patient or Public Contribution:**

No patient or public contribution was applicable, as the study focused on nurses' experiences.

## 1 Introduction

Emotional demands and emotion regulation, individually and in combination, have been linked to burnout and poorer mental health among nurses. Nurses frequently encounter work‐related emotional demands, including emotionally charged situations, challenging interactions with patients and relatives and experiences of moral distress (Oldenmenger et al. 2024; Popucza et al. 2025). Meeting these demands may require nurses to regulate their emotions and emotional expressions in accordance with professional role expectations (Feng et al. 2024). Exposure to emotional demands and the regulation of emotions at work have been associated with poorer health and well‐being and may contribute to challenges in sustaining and retaining the nursing workforce (Feng et al. 2024; Oldenmenger et al. 2024; Popucza et al. 2025).

Although meta‐analytic evidence suggests that older nurses report slightly lower levels of burnout compared with younger nurses, these effects are small and highly heterogeneous, and the overall evidence remains inconclusive (Gómez‐Urquiza et al. 2017). Findings across reviews and large empirical studies vary substantially, with some studies reporting age‐related associations with burnout, whereas others report no significant age differences (Adriaenssens et al. 2015), and more recent evidence associating older age with higher levels of burnout on the dimensions of emotional exhaustion and depersonalization (Getie et al. 2025).

While the relationship between age, burnout and nurses' health and well‐being appears complex, one theoretical perspective that has been used to interpret potential age‐related differences in emotional processes is Socioemotional Selectivity Theory (SST) (Carstensen et al. 1999). SST proposes that changing motivational priorities across adulthood may contribute to improved emotional well‐being later in life. Drawing on this perspective, a substantial body of work has proposed that age may be associated with advantages in emotional functioning at work, including a conceptual overview and framework by Scheibe et al. (2021). They synthesized lifespan theories and empirical research suggesting potential age‐related strengths in emotional experience, emotion regulation and emotional competencies, while also emphasizing contextual boundary conditions and potential vulnerabilities. However, a recent systematic review found that empirical evidence for age‐related differences in emotional work demands and emotion‐regulatory strategies is inconsistent, and that studies invoking SST rarely directly assess its core mechanisms, such as perceived time horizons or changing goal priorities (Popucza et al. 2026). Recent empirical studies likewise found little evidence that age consistently influences associations between emotional demands, emotional labour, moral distress and burnout (Popucza et al. 2025; Popucza and Eriksson 2026). Taken together, the available evidence does not support a simple or consistent age‐related pattern in emotional demands or emotion regulation at work. However, little is known about how nurses themselves describe changes in emotional demands and emotion regulation across long nursing careers. Accordingly, the present study uses SST as an interpretive perspective for contextualizing potential age‐related patterns rather than as a theoretical framework guiding the study design or analysis.

In this study, emotional demands and emotion regulation are conceptualized as related but distinct aspects of emotional work. Emotional demands refer to work‐related exposures involving emotionally taxing situations or requirements, while emotion regulation is used here as a broad term encompassing processes and strategies through which individuals manage emotional experience or expression in relation to such emotionally challenging work situations, including emotional labour, understood here as the management of emotional experience or expression in accordance with professional role expectations (Popucza et al. 2026). Emotion regulation may include cognitive, expressive, interpersonal or behavioural strategies used during or following such situations to influence emotional experience or expression (Gross 1998; Koole 2009; Zaki and Williams 2013). Emotion regulation was therefore understood functionally, as strategies used to influence emotional experience or expression in response to emotionally challenging situations. It was explored broadly and was not restricted to a predefined set of cognitive, expressive, interpersonal or behavioural strategies. These concepts were used to delimit the phenomena of interest and inform the interview guide rather than as predefined analytical categories.

The present study was conducted within the Swedish publicly funded healthcare system. Like many healthcare systems internationally, the Swedish healthcare system has in recent decades been characterized by workforce shortages, rising care demands and efficiency pressures. Within such healthcare contexts, emotional demands and emotion regulation are important factors associated with nurses' health and well‐being and have been linked to burnout, premature exit from the profession, and sustainable workforce retention. Understanding how late‐career nurses describe changes in emotional demands and emotion regulation across their careers may provide insight into what these changes entail and how they are experienced and understood over time. Such knowledge may help clarify inconsistent age‐related findings in previous research, inform theoretical refinement and future longitudinal research, and contribute to organizational policies and practices supporting sustainable participation across the working lifespan. A recent systematic review on emotional work demands and emotion‐regulatory strategies highlighted that the older segments of the workforce remain underrepresented in this research, with workplace studies frequently defining ‘older’ workers at substantially younger ages than those typically examined in the broader literature on emotions and ageing (Popucza et al. 2026). Focusing on nurses aged 60 years and older therefore enabled the present study to include workers more closely corresponding to later‐adulthood samples in the emotions and ageing literature while also capturing nurses in the later stages of working life. This is particularly relevant in Sweden, where retirement timing is flexible, and employment can extend beyond age 60. Accordingly, this study aims to explore how late‐career nurses aged 60 years and older, with at least 20 years of nursing experience, describe changes in emotional demands and emotion regulation throughout their careers.

## 2 Method

### 2.1 Design

A qualitative interview study using qualitative content analysis was conducted. The study had a primarily descriptive qualitative orientation and was not guided by a specific philosophical research paradigm. Qualitative content analysis was selected because it allows systematic analysis of similarities and differences in interview data and can be used to develop descriptive categories at varying levels of abstraction while remaining grounded in the participants' accounts (Graneheim and Lundman 2004; Graneheim et al. 2017; Lindgren et al. 2020). The study adhered to the Standards for Reporting Qualitative Research (SRQR), supporting transparency and methodological clarity (O'Brien et al. 2014). The completed SRQR checklist is provided as Data S1.

### 2.2 Participants

Participants were recruited through self‐referral following a study advertisement posted on Facebook. The advertisement was promoted to Facebook users across Sweden and described the study together with the eligibility criteria: (a) registered nurse qualification, (b) age 60 years or older, (c) a minimum of 20 years of professional nursing experience, (d) current employment of at least 50% of full‐time, and (e) current work including direct patient care. In total, 44 individuals expressed interest in participating. Of these, 16 did not meet the inclusion criteria and were excluded. The remaining 28 eligible individuals were invited to participate in two recruitment rounds. Interviews were scheduled with all eligible individuals who responded to the invitation or subsequent reminders and could be booked during the data collection period. Thirteen interviews were ultimately conducted. One eligible individual responded after data collection had been completed and analysis had begun and was therefore not included. The remaining eligible individuals did not respond to the invitation or reminders, or an interview could not be scheduled during the data collection period. No participants withdrew after agreeing to participate. Participant characteristics are presented in Table 1.

**TABLE 1:** Participant characteristics.

Characteristic	Description
Participants	13
Age, years	60–70
Gender	12 women; 1 man
Professional nursing experience, years	25–48
Current employment	All participants were employed at ≥ 50% of full‐time
Current patient care	All participants' current work included direct patient care
Professional roles and employment arrangements	Registered nurses, specialist nurses, one midwife, nurse educators, nurse managers/coordinators and agency nurses
Clinical settings represented	Emergency care, intensive care, anaesthesia, radiology, surgical and urological care, peritoneal dialysis, maternity care, residential aged care, patient hotel care and outpatient care.

*Note:* Professional roles and clinical settings refer to participants' employment at the time of the interviews.

### 2.3 Data Collection

Data collection was conducted as part of a larger research project comprising two data‐collection components: a large‐scale survey and a qualitative interview component. The present study reports the qualitative component and constitutes a primary analysis of these interview data. Participants were recruited through an advertisement on the University's official Facebook page. Interested individuals completed an online expression‐of‐interest form that included screening questions regarding age, profession, length of work experience, and current work situation. Responses were assessed against the study's inclusion criteria. Eligibility and current work situation were reconfirmed at the beginning of each interview. All interviews were conducted by the first author. The interviewer had no prior relationship with the participants before recruitment.

A total of 13 individual interviews were conducted between 29 August and 15 October 2025 using a semi‐structured interview guide. The semi‐structured interview guide was developed to address the study aim and was informed by previous research on emotional demands, emotional labour, and emotion regulation, rather than by a predetermined theoretical framework. It included questions such as: ‘Can you think of situations that you experience as emotionally challenging?’, ‘When you encounter such emotional demands, that is, emotionally challenging experiences, how do you maintain your professional role?’, and ‘Have you noticed any changes over the course of your nursing career in how you responded to emotionally challenging experiences and maintained your professional role, or has it remained the same?’. The questions included in the semi‐structured interview guide, including the descriptions and examples used to explain the phenomena of interest, were asked of all participants in the same sequence. Additional follow‐up questions were formulated flexibly during the interviews to encourage elaboration and clarification, for example by asking participants to recall another situation, provide an additional example, consider whether anything else came to mind, or elaborate further on their response. The English translation of the interview questions, including how the phenomena of interest were presented and explained to participants, is provided in Data S2. The interview guide was pilot‐tested with one retired nurse with extensive professional experience. Following the pilot interview, minor revisions were made to improve clarity. The pilot interview was not included in the analysis.

All interviews were conducted in Swedish and held online via Zoom or Microsoft Teams. Interviews were audio‐recorded and had an average duration of approximately 50 min (range: 34–70 min). Audio recordings were transcribed using automated transcription software (Amberscript 2025). All transcripts were subsequently checked and corrected against the audio recordings to ensure accuracy. Data analysis commenced after all interviews had been completed. Consequently, sampling and the interview guide were not iteratively modified in response to emerging findings. This approach ensured consistency across interviews and minimized the influence of preliminary analytic interpretations on subsequent data collection. The verified transcripts constituted the empirical material for the study.

### 2.4 Data Analysis

Qualitative content analysis was used to analyze the interview data (Graneheim et al. 2017; Lindgren et al. 2020). The analysis remained close to participants' descriptions while involving increasing abstraction during category development. In qualitative content analysis, abstraction and interpretation are distinct dimensions, and descriptive categories may be developed at higher levels of abstraction without necessarily representing interpretations of latent content (Graneheim et al. 2017; Lindgren et al. 2020). A relatively low degree of interpretation was maintained throughout condensation, coding and category development, so that the analysis remained predominantly descriptive rather than focused on underlying latent meanings. All transcripts were read repeatedly to achieve familiarization with the data. Meaning units relevant to the study aim were identified and condensed in Swedish. The first author then generated English‐language codes directly from the condensed Swedish meaning units. The coding process and the correspondence between the condensed meaning units and the English codes were reviewed and discussed with the second author before further analysis.

A total of 381 codes were then manually sorted according to similarities and differences in content to support the initial exploration of patterns and relationships within the data. Based on these preliminary groupings, the codes were subsequently organized using Microsoft Word and Excel and repeatedly regrouped, compared, merged and refined to develop increasingly coherent categories. Throughout this process, the evolving analytic structure was continuously checked against the condensed meaning units and, where needed, the original interview transcripts to ensure that the emerging groupings remained grounded in participants' descriptions and their original context. This iterative process continued until the final categories and hierarchy of categories were established. All 13 participants contributed data to the main categories Professional and Personal Dimensions and Workplace Features, while 12 participants contributed data to Societal Transformations.

The first author conducted the primary analysis, while analytic decisions, category development, and category naming were continuously discussed with the second author throughout the analytic process. Any uncertainties regarding coding or category placement were resolved through discussion between the authors, considering the code, the condensed and original meaning units, and where necessary, the original transcript for contextual understanding, to clarify similarities and differences in the developing categories. An illustrative example of the analytic process, from condensed meaning unit to code, category and main category, is presented in Data S3.

Illustrative quotations were selected to represent variation across participants and categories, while also considering the clarity with which participants articulated their experiences. Participant quotations were translated into English only after the analysis had been completed. The translations were prepared by the first author and reviewed by the second author, both of whom are proficient in Swedish and English, to ensure that the English quotations accurately reflected the meaning and tone of the original Swedish expressions. Quotations are identified by anonymous participant numbers (P1–P13), corresponding to the order in which the interviews were conducted.

### 2.5 Rigour

Rigour was addressed through strategies enhancing credibility, dependability and transferability, in line with established criteria for qualitative content analysis (Graneheim et al. 2017). Credibility was supported through attention to sample adequacy and carefully considered data collection and analytic choices. Information‐power considerations were used to inform judgments of sample adequacy during data collection, considering the focused research aim, the specificity of the sample, and the rich and relevant dialogue generated through the semi‐structured interviews (Malterud et al. 2016; Vasileiou et al. 2018). Data collection was concluded after 13 interviews, when later interviews were judged to be largely reiterating information already raised, with relatively little new information emerging. The final sample of 13 participants also falls within empirically observed ranges in qualitative interview research and is consistent with broader methodological guidance for focused qualitative studies (Hennink and Kaiser 2022; Vasileiou et al. 2018). No concurrent formal coding or analysis was conducted during data collection. The semi‐structured interview guide was used consistently across interviews, together with a systematic and transparent analytic procedure. A close connection to participants' descriptions was maintained throughout the analytic process while allowing increasing abstraction during category development. Evolving categories were repeatedly checked against condensed meaning units and, when needed, the original transcripts to maintain grounding in the data (Graneheim et al. 2017; Lindgren et al. 2020). Dependability was supported through consistent interview procedures, detailed documentation of data collection and analysis, including successive versions of the developing analytic structure, and continuous review of analytic decisions. Analytic decisions were discussed among the authors, and the evolving analytic structure was continuously reviewed to enhance consistency over time (Lindgren et al. 2020). Formal analysis commenced approximately three weeks after completion of the interviews and was conducted over approximately two months. Because analysis began only after data collection was complete, emerging analytic interpretations did not influence subsequent interviews or lead to changes in the interview guide, supporting consistency in data collection across participants. Transferability was addressed by providing clear descriptions of the study context, participant characteristics and data collection procedures, enabling readers to assess the relevance of the findings to other settings (Graneheim et al. 2017). Reflexivity was addressed by recognizing that the researchers brought complementary disciplinary and professional perspectives to the study. The first author has a background in emotion psychology and occupational health science, with research focusing on emotions at work. This perspective informed the conceptual framing and the development of the interview guide, including the focus on emotional demands, emotion regulation and changes across the nursing career. The second author has a background in nursing, care sciences, qualitative research, and qualitative content analysis, providing a complementary nursing and methodological perspective during the analysis. No repeat interviews or participant validation were conducted; transcripts or findings were not returned to participants, and no formal field notes were created during the interviews or used in the analysis.

### 2.6 Ethical Considerations

The study was conducted in accordance with national ethical regulations and established principles for responsible research, as outlined in the ALLEA European Code of Conduct for Research Integrity (ALLEA 2023). Ethical approval was obtained from the Swedish Ethical Review Authority (Dnr: 2023‐08117‐01). All participants received written and verbal information about the study's aim, procedures, voluntary nature of participation and the confidential handling of data. Participants were informed of their right to withdraw at any time without consequences. Informed consent was obtained orally from all participants before the interviews and was audio‐recorded. All data were handled and stored in accordance with data protection regulations to ensure participant confidentiality. Digital data processing, including transcription, was conducted using services approved for institutional use and in accordance with the University's data‐protection procedures and applicable data‐processing agreements. Participant characteristics are presented in a way that minimizes the risk of participant identification. Findings are reported at the group level, and quotations are presented without details that could enable the identification of individual participants.

### 2.7 Use of AI

Automated transcription software was used to transcribe the interview recordings (Amberscript 2025). All transcripts were subsequently checked and corrected by the researchers through comparison with the original audio files to ensure accuracy. Generative AI tools, including ChatGPT (OpenAI 2026), were used solely for language refinement and editing of the manuscript. All AI‐assisted revisions were carefully reviewed and approved by the authors, who retained full responsibility for the final wording and scientific content of the manuscript. No generative AI tools were used for data analysis, interpretation of findings, or generation of results or conclusions.

## 3 Results

This study explored how thirteen nurses aged 60 years and older, with at least 20 years of nursing experience, described changes in emotional demands and emotion regulation throughout their careers. Throughout the Results, participants' accounts were described as emotional demands when they concerned emotionally taxing work situations or exposures, and as emotion regulation when they concerned managing emotional experience or expression in response to such situations. Other findings were presented as conditions shaping emotional demands or opportunities for emotion regulation. The results are organized into three main categories: Professional and Personal Dimensions, Workplace Features and Societal Transformations, with their corresponding categories. The labels assigned to the main categories and corresponding categories were analytic constructions developed during the qualitative content analysis rather than participants' own terminology. Figure 1 presents the empirical category structure derived from the qualitative content analysis, illustrating the analytical hierarchy of the three main categories and their corresponding categories.

**FIGURE 1:**
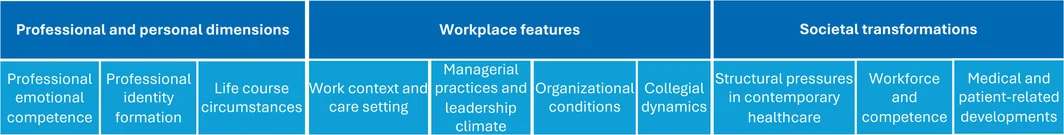
Analytical hierarchy of the categories derived from the qualitative content analysis.

### 3.1 Professional and Personal Dimensions

This main category represents participants' descriptions of changes in emotional demands and emotion regulation in relation to professional and personal dimensions throughout their nursing careers. Participants described these changes as emerging from the development of professional emotional competence, the formation of professional identity and changing circumstances throughout their life course.

### 3.2 Professional Emotional Competence

Participants described professional emotional competence as developing through increased preparedness, confidence and increasingly deliberate approaches to managing emotionally demanding situations throughout their nursing careers. Many participants described their early professional years as marked by strong emotional reactions, efforts to conceal emotions to appear professional and competent, a tendency to carry emotionally demanding experiences beyond the workplace and private emotional processing after work, often amid limited confidence and support.

Over time, leaving work‐related emotions at work was described as becoming easier and important for sustaining long‐term engagement in nursing. Looking back across their careers, several participants described becoming calmer (e.g., ‘Emotions don't go up and down in the same way as they did when I was younger’ [P1]) and more confident in emotionally demanding situations. Some participants described repeated exposure as making similar situations feel more familiar and manageable over time. Many participants described improved emotional control and clearer boundaries between professional and private life, while some intense emotional situations remained challenging to manage, for example, as one participant explained: ‘If it's a very sensitive situation…//…and it affects me a lot, a tear may come even for me, and that should be okay’ (P9).

While emotional concealment remained an important professional skill, many participants described greater openness in expressing care and compassion when appropriate. Several participants also described learning to remain present and empathetic in sensitive encounters while postponing emotional processing. Emotion regulation strategies were sometimes described as shifting from unreflective endurance toward more conscious and intentional approaches, including reflective communication, reframing, compartmentalization, seeking collegial support and strategies for recovering from work‐related emotional strain.

Together, these findings illustrate how participants described changes in emotional demands and emotion regulation throughout their nursing careers in relation to the development of professional emotional competence, characterized by increasing preparedness, confidence and increasingly deliberate approaches to managing emotionally demanding situations while remaining emotionally engaged in patient care.

### 3.3 Professional Identity Formation

Participants described the formation of their professional identity as being shaped by emotionally significant experiences, reflection and meaning‐making, and an increasing sense of responsibility to support less experienced colleagues. Together, these findings illustrate how participants described the formation of their professional identity throughout their nursing careers.

Participants described specific emotionally intense events, including repeated exposure to patient death, traumatic incidents, ethical conflicts, boundary transgressions, witnessing unprofessional or disrespectful care, and experiences of inadequate organizational support, as leaving lasting impressions that shaped how they approached their work as nurses. Such experiences were often described as prompting changes in behavior, professional role orientation, or career direction. One participant reflected on an experience that led to leaving a previous role: ‘I couldn't handle it. And I felt that I had to quit’ (P10). Participants described these experiences as having contributed over time to heightened emotional sensitivity, clearer professional boundaries, and increased confidence in addressing ethically challenging situations.

Participants further described how reflection on emotionally demanding experiences contributed to a growing sense of meaning in their work and reinforced their understanding of their core professional values. Patient encounters, including work in long‐term care, psychiatry, palliative care, emergency care and cancer care, were described as emotionally demanding while simultaneously strengthening participants' understanding of nursing, human vulnerability and professional responsibility. As one participant explained, ‘the work with severely ill cancer patients I found to be a fantastic assignment…//…it was so meaningful in many ways’ (P8). Meaning‐making was often described as developing gradually through experience and reflection rather than through deliberate effort.

Participants also described an increasing responsibility to support and guide less experienced colleagues as an important aspect of their professional identity. Several participants explained how they had themselves learned from both positive and negative role models earlier in their careers and described passing these experiences and professional values on to others. Acting as mentors, role models and patient advocates, supporting colleagues in emotionally demanding situations, and encouraging reflection were described as ways of contributing to the professional development of others while maintaining standards of compassionate and ethical nursing practice.

Together, these findings illustrate how participants described changes in emotional demands and emotion regulation throughout their nursing careers in relation to the formation of their professional identity through emotionally significant experiences, reflection and meaning‐making, and the transmission of professional values and emotional competence.

### 3.4 Life Course Circumstances

Participants described changes in emotional demands and emotion regulation throughout their nursing careers in relation to changing life course circumstances outside and alongside work, including family responsibilities, recovery needs, career transitions and changing personal resources. Earlier in life, emotional strain from work was often managed alongside extensive family responsibilities, leaving limited time and energy for emotional and physical recovery. Caring for children in combination with demanding work was often described as restricting recovery and, in some cases, as coinciding with accumulated stress, exhaustion or periods of burnout. One participant illustrated these differences by reflecting: ‘It depends on your life situation. Back then, you didn't have the possibility to come home from work the way you can now. I can take a walk; I can have a cup of coffee and sit down for a while. It was about life circumstances and what kind of family situation you were in when you had small children’ (P12).

Ways of processing emotionally demanding experiences were also described as changing over time. Earlier in life, recovery was often described as occurring through family life, social activities, or conversations with partners, whereas later in life, several participants described more selective use of support and less need for deliberate emotional recovery. While several participants described having greater opportunities for recovery as family responsibilities changed, they also described an increased need for physical rest and sleep later in their careers. A few participants also described new emotional challenges associated with being older in the workplace, including compassion fatigue following prolonged exposure to emotionally demanding work, and an increased sensitivity to age‐related criticism, for example, feeling that their competence was questioned, or experiencing diminished respect because of their age. As one participant reflected, ‘…when you're questioned…//… [by] new or relatively new doctors who think they know everything…//… [who are] condescending…//… and don't listen to the experience and competence that nurses have…//… then I think, “I don't have to put up with that.”’ (P7). For some participants, self‐chosen career transitions were described as responses to changing personal capacities, tolerance and private‐life demands, supporting continued participation in working life, while accommodating changes in energy, health and personal priorities.

Together, these findings illustrate how participants described changes in emotional demands and emotion regulation in relation to changing life course circumstances through adaptations to shifting family responsibilities, personal resources, recovery needs, and career priorities throughout their nursing careers.

### 3.5 Workplace Features

This main category represents participants' descriptions of changes in emotional demands and emotion regulation in relation to varying workplace features throughout their nursing careers. Participants described workplace features in terms of different work contexts and care settings, managerial practices and leadership, organizational conditions, and collegial dynamics. These categories capture analytically distinct aspects of the immediate work environment and changes in these aspects across participants' careers. Although presented separately, these workplace features were often described as interconnected and together shaped both emotional demands and opportunities for emotion regulation within the immediate work environment.

### 3.6 Work Context and Care Setting

Participants described emotional demands and emotion regulation as varying across different work contexts and care settings, reflecting differences in patient encounters, clinical responsibilities, and exposure to emotionally demanding situations. Many participants described roles involving acute care, intensive care, cancer care and home‐based palliative care as emotionally demanding due to frequent exposure to traumatic situations, patient death, unpredictable events and close involvement with patients and relatives, sometimes involving personal grieving. One participant described this: ‘Over all these years, I have worked a lot in cancer care. I've had hundreds of patients who have died. So of course, there are a lot of emotions in the work’ (P1).

In contrast, several participants described planned and predictable care settings as less emotionally demanding. Some participants explained that planned care reduced acute emotional strain because unexpected events occurred less frequently, for example: ‘everything is planned. Nothing acute ever comes up to interrupt things, so that's probably the difference’ (P2). Shorter patient encounters and reduced exposure to relatives' distress were described as limiting emotional involvement. However, several participants described that daily contact with seriously ill patients and responding to patients' fears remained emotionally demanding regardless of the care setting. Participants further described how specific characteristics of the clinical role influenced emotional demands. Individual participants described aggressive or threatening patient behaviours as particularly difficult to manage emotionally, whereas greater decision‐making autonomy in some roles was described as facilitating better management of time, priorities, and emotionally demanding situations.

Together, these findings illustrate how participants described changes in emotional demands and emotion regulation in relation to different work contexts and care settings, shaped by the nature of patient encounters, clinical responsibilities and exposure to emotionally demanding situations.

### 3.7 Managerial Practices and Leadership Climate

Participants described variations in managerial practices and leadership climate as shaping emotional demands and opportunities for emotion regulation through leadership support, managerial presence, ethical awareness and responses to emotionally challenging situations throughout their nursing careers. Many participants described how supportive leadership, characterized by availability, openness to dialogue and ethical awareness, facilitated emotional processing and the management of emotionally challenging experiences. In contrast, lack of managerial support, limited presence, and inadequate follow‐up after serious incidents were described as major sources of emotional strain. Some participants described experiences of poor leadership as more emotionally burdensome than patient care itself. For example, one participant said, ‘It is much more the managers and the leadership that have upset me than the work itself’ (P1). Some participants also described changes in managerial practices over time, including increased economic focus and reduced clinical understanding among managers, as intensifying emotional strain. Additionally, several participants described managers whom they perceived as lacking insight into nursing work and as imposing unrealistic work demands.

In some participants' accounts, reporting abuse, neglect or ethical concerns without receiving leadership support or meaningful follow‐up was described as particularly emotionally difficult and, for some participants, associated with persistent moral distress. As one participant explained: ‘it was swept under the carpet at every level…//…It feels heavy when I think about it’ (P8). In addition, when some participants themselves held managerial roles, they described emotional burdens related to responsibility, confidentiality and decision‐making pressure, often involving solitary emotional processing, while access to professional support was described as helpful when available.

Together, these findings illustrate how participants described variations in managerial practices and leadership climate as shaping emotional demands and opportunities for emotion regulation through leadership support, managerial presence, ethical awareness and responses to emotionally challenging situations while also highlighting the emotional demands associated with leadership responsibilities.

### 3.8 Organizational Conditions

Participants described varying organizational conditions within their immediate workplaces as shaping emotional demands and opportunities for emotion regulation through formal and practical aspects of the work environment, including working conditions, organizational support, opportunities for emotional reflection, and available resources throughout their nursing careers. Many participants described the absence of organizational support, acknowledgment, and structured opportunities for emotional reflection as leaving emotional processing largely to individuals or informal collegial support. Lack of regular and structured support was commonly described, alongside a normalization of emotional suppression and a persistent carry‐on culture. In contrast, structured reflection and debriefing were described by some participants as supportive and by others as something they wished had been available more regularly. As one participant reflected: ‘It would be nice, perhaps, if one could have recurring debriefings’ (P13).

Workload, work pace, staffing levels, work hours and employer treatment were described by many participants as major sources of emotional strain, in some cases described as more distressing than patient‐related demands. Inability to provide safe care due to overwhelming workload was described by several participants as causing emotional distress and safety concerns. In contrast, calm work environments and a manageable work pace were sometimes described as supporting emotional regulation and professional security. As one participant explained: ‘it is of course easier to keep your calm when it is calm [at work]’ (P1). Some participants also described a lack of organizational recognition and empathy toward staff as particularly emotionally burdensome and, in some cases, associated with long‐term stress, sick leave, or decisions to leave workplaces.

Individual participants also described ongoing or recurring organizational restructurings, changes in organizational culture, and differences between urban and rural healthcare contexts as shaping the work environment and the emotional climate of the workplace. A few participants also described working evenings and weekends, particularly when staffing was limited, as emotionally demanding because difficult decisions often had to be made with limited support.

Together, these findings illustrate how participants described varying organizational conditions as shaping emotional demands and opportunities for emotion regulation by influencing the support, resources, and working conditions available within their workplaces throughout their nursing careers.

### 3.9 Collegial Dynamics

Participants described collegial dynamics within their immediate workplaces as shaping emotional demands and opportunities for emotion regulation through interpersonal relationships and team‐level processes, including emotional support, shared reflection, trust, and psychological safety throughout their nursing careers. As one participant explained, ‘now I have a nursing assistant with me all the time, so we help each other…//… and that feels really good because when I feel like I'm about to break…//… then I can step outside and talk to my colleagues’ (P13). Supportive collegial climates, characterized by trust, shared experience, and openness, were described as enabling emotional expression, shared reflection, and processing of emotionally demanding situations. Working in stable teams with experienced colleagues was described as fostering emotional security and ease in seeking support, for example: ‘In the past, when I first started working in the delivery ward, you always worked with the same people. So, when I was in a delivery, I sometimes only needed to look at my nursing assistant, and she knew exactly what I wanted’ (P13).

Most participants described great variation in collegial climates across work environments. Stable and experienced teams with trusting communication were often described as reducing emotional strain, whereas high staff turnover, lack of experienced colleagues and poor group dynamics were sometimes described as undermining emotional security. In such contexts, several participants described relying more on emotional support outside the workplace. Collegial tensions, cliques and role‐related conflicts were described by some participants as creating emotionally uncomfortable situations and, in some cases, as contributing to decisions to leave workplaces.

Participants also described how collegial dynamics could both buffer and intensify other emotional burdens. Many participants described collegial support as helping manage frustration related to leadership and organizational conditions, while poor team climate and accumulated disillusionment among colleagues were sometimes described as emotionally demanding in themselves. Many participants described opportunities to express emotions as dependent on shared group norms, with experienced nurses sometimes describing efforts to encourage reflection or model emotional openness within teams.

Together, these findings illustrate how participants described the varying collegial dynamics shaping emotional demands and opportunities for emotion regulation through emotional support, trust, shared reflection and the social climate within work teams throughout their nursing careers.

### 3.10 Societal Transformations

This main category represents participants' descriptions of changes in emotional demands and emotion regulation in relation to broader societal transformations throughout their nursing careers. Participants described different aspects of broader change, including system‐level pressures in healthcare, changes in the characteristics of the healthcare workforce, and developments in medical care, technology and patient populations as reshaping the conditions under which nursing work was carried out.

### 3.11 Structural Pressures in Contemporary Healthcare

Participants described broader changes extending beyond the immediate workplace, reflecting pressures and developments in the wider healthcare system, as shaping emotional demands and opportunities for emotion regulation through changes in healthcare governance, administrative demands, resource availability and organizational priorities throughout their nursing careers. As one participant reflected, ‘Healthcare in the public sector…//… is under such economic, legal, and political pressure’ (P6). Several participants described shifts toward greater administrative and economic control as reducing professional autonomy, narrowing the scope of nursing work, and increasing workload. Many participants described reduced resources, administrative delays, and longer decision‐making processes as contributing to patient safety concerns and experiences of stress, frustration and moral distress.

Several participants described nurse shortages, reliance on temporary agency staff, and shortages of experienced personnel as intensifying workload for permanently employed nurses. Many participants described a faster work pace, rising performance demands and extensive documentation as central sources of emotional strain, alongside feelings of constantly falling behind, for example: ‘it can be emotionally challenging to go to work, and you do your best, but you never catch up, and you constantly feel that you are behind’ (P2). Many participants also described that administrative and digital demands were both reducing time available for patient interaction and care and intensifying emotional strain.

A few participants also described how these structural pressures influenced the perceived value of nursing work and the ability to provide good care. In individual participants' accounts, the perceived devaluation of nursing work evoked strong emotional and moral reactions. Others described the accelerated pace of contemporary healthcare as increasingly difficult to accommodate in later working life, particularly when workplace adaptations were perceived as insufficient to meet changing needs later in their careers. A few participants also described emotional distress related to inadequate elder care, end‐of‐life suffering, and situations in which adequate care could not be provided.

Together, these findings illustrate how participants described structural pressures in contemporary healthcare as influencing emotional demands and opportunities for emotion regulation through changes in healthcare governance, administrative demands, organizational priorities and the resources available to support nursing care throughout their nursing careers.

### 3.12 Workforce and Competence

Participants described broader changes extending beyond the immediate workplace, reflecting changes in the wider healthcare workforce, as shaping emotional demands and opportunities for emotion regulation through workforce composition, professional competence, communication and professional values throughout their nursing careers. Compared with earlier in their careers, several participants described greater openness among staff to ask questions, alongside increased questioning of authority and reduced respect for professional experience, which they described as making supervisory and leadership responsibilities more emotionally demanding. A few participants described intergenerational differences as shaping communication and expectations at work and described uncertainty about how to communicate without offending, sometimes leading them to choose emotional restraint. Some participants also described feeling less respected by younger colleagues and experiencing what they perceived as shifts away from collective responsibility, contributing to feelings of isolation among experienced nurses seeking to uphold shared professional standards.

Some participants also described what they perceived as reduced professional competence, empathy, and mutual trust among staff as increasing emotional strain for experienced nurses. Several participants described high staff turnover and workforce instability (e.g., ‘I see new people at work every single day’ [P8]) and language‐related communication difficulties (e.g., ‘there are major language difficulties’ [P8]) as intensifying the need for guidance and supervision. Some participants also described heightened responsibility to intervene and uphold standards of professional and compassionate care, for example, when witnessing inappropriate behaviour toward vulnerable patients: ‘If a staff member stands and shouts at a person with dementia, then you simply haven't understood’ (P10).

Some participants further described changes in professional hierarchies and collaboration patterns, increased control‐oriented approaches, and unrealistic expectations regarding skills, experience, and workforce readiness, as overlooking the importance of accumulated professional maturity and contributing to frustration among experienced nurses.

Together, these findings illustrate how participants described changes in the healthcare workforce as influencing emotional demands and opportunities for emotion regulation through workforce composition, professional competence, communication and professional values throughout their nursing careers.

### 3.13 Medical and Patient‐Related Developments

Participants described medical and patient‐related developments as shaping emotional demands and opportunities for emotion regulation through advances in medical care and technology, changing patient populations, and changing patient expectations throughout their nursing careers. Many participants described advances in medical treatment and technology as improving patients' chances of survival, reducing some of the emotional demands associated with patient deaths. At the same time, improved survival was described as contributing to increasingly complex care situations. As one participant reflected, ‘I feel that I am faced with more complicated situations now, with more advanced care’ (P3). Several participants described improved monitoring and technical development as enhancing safety in some areas. However, they were also described as increasing workload, responsibility and pressure to perform correctly.

Several participants described increased emphasis on documentation and digital systems as reducing time for direct patient interaction as well as conversations with colleagues. This was sometimes described as reducing understanding of patients' situations and opportunities to develop shared contextual knowledge among colleagues. While some participants described a shift toward a more patient‐centred approach to care, with technology viewed as supportive rather than dominant, others described increasing technical emphasis at the expense of caregiving, leading to a colder work culture.

Changes in patient characteristics and expectations were described as further intensifying emotional demands. Several participants described changes in patients and relatives, including increased access to online information, greater autonomy and higher expectations, which several participants described as increasing emotional and moral challenges related to communication, boundary‐setting, and responsibility. One participant reflected on how increased patient autonomy could complicate care decisions: ‘Patients today also want…want to decide so much themselves…//…in ways that don't benefit them…//…so they are perhaps a bit more confrontational, maybe to their own disadvantage’ (P13).

Broader changes in patient populations were also described as affecting emotional demands. Some participants described increased patient anxiety, reduced self‐reliance and more frequent encounters with mental illness, violence‐related injuries and suicides as emotionally challenging. Some participants also described changes over time in the frequency and types of injuries associated with specific occupational, recreational or transportation‐related accidents. Several participants described working with older patients with complex needs and limited time for care and communication as further increasing emotional strain and moral distress. A few participants described what they perceived as differences in communication and emotional expression among patients and relatives from different cultural backgrounds as sometimes making emotionally demanding encounters more difficult to manage while maintaining a professional role.

Together, these findings illustrate how participants described medical and patient‐related developments as shaping emotional demands and opportunities for emotion regulation through advances in medical care and technology, changing patient populations and changing patient expectations throughout their nursing careers.

## 4 Discussion

This study explored how late‐career nurses described changes in emotional demands and emotion regulation across nursing careers spanning 20 years or more. Participants described emotional demands as persisting throughout their careers, while changes in how these demands were experienced and regulated were attributed to the intersection of professional and personal dimensions, workplace features and broader societal transformations, rather than to chronological age alone. The findings are discussed in relation to previous research, with Socioemotional Selectivity Theory used as an interpretive perspective for contextualizing potential age‐related patterns in emotional processes. Subsequent sections consider the findings in relation to lifespan transitions as processes of recalibration rather than linear development, professional identity formation in emotionally demanding work, the cumulative and organizationally embedded nature of emotional demands, and broader societal transformations shaping contemporary nursing practice. Together, these perspectives suggest that participants' descriptions of changes in emotional demands and emotion regulation across their nursing careers reflect dynamic, contextual and cumulative processes that cannot be understood solely in relation to chronological age.

### 4.1 Age and Emotion Regulation: A Socioemotional Selectivity Theory Perspective

SST (Carstensen et al. 1999) has frequently been used to interpret age‐related differences in emotion regulation at work, with age‐related motivational shifts proposed as one explanation for potentially more favourable emotional functioning and work‐related outcomes among older workers (e.g., Scheibe et al. 2021). Recent clarifications of SST, however, emphasize that age‐related motivational shifts concern the prioritization of emotionally meaningful goals in response to changing perceived time horizons, rather than enhanced strategic regulation of emotional states in the moment (Carstensen et al. 2024). From this perspective, SST does not imply that emotionally taxing work environments become less emotionally demanding with age.

While the present study did not directly assess perceived time horizons, changing goal priorities, or other mechanisms central to SST, SST may still offer an interpretive perspective for considering potential age‐related patterns in emotional functioning at work. Participants' accounts suggest that chronological age alone may be insufficient to explain perceived changes in emotional demands and regulation across long nursing careers, consistent with recent systematic evidence showing no consistent age‐related differences in emotional work demands or emotion‐regulatory strategies (Popucza et al. 2026). Although participants described increased confidence, selectivity and intentionality in emotional engagement over time, they also reported persistent and cumulative emotional strain and continued sensitivity to organizational and moral stressors in later career stages. These accounts complicate simplified applications of lifespan theories in workplace contexts that are sometimes taken to imply systematic age‐related improvements in emotional functioning, despite theoretical emphasis on contextual boundary conditions and vulnerabilities (Charles 2010; Scheibe et al. 2021). Rather than indicating a uniform developmental trajectory, participants described changes as emerging through the intersection of professional and personal dimensions, workplace features, and broader societal transformations. These descriptions suggest that changes in emotional demands and emotion regulation in late‐career nursing reflect the development of professional emotional competence and professional identity, alongside changing life course circumstances, and are further shaped by workplace context, leadership, organizational conditions, collegial dynamics and broader societal changes related to the workforce, patient characteristics and medical advances, and pressures within healthcare, rather than by age‐related motivational shifts alone.

### 4.2 Lifespan Transitions: Recalibration, Not Linear Improvement

Participants' accounts can be interpreted as reflecting an ongoing process of recalibration rather than linear improvement with increasing age. Participants described continually adapting to changing personal circumstances, evolving professional roles, different workplace contexts, and broader changes in healthcare, requiring ongoing adjustments in how emotionally demanding work was experienced and managed. This interpretation is consistent with recent systematic evidence showing no generalizable pattern of age‐related differences in emotional work demands or emotion‐regulatory strategies, with findings varying across contexts, measures, and analytical approaches (Popucza et al. 2026). Similarly, previous research on burnout has shown weak, inconsistent or context‐dependent associations with age (Gómez‐Urquiza et al. 2017), further supporting caution against interpreting emotional vulnerability as changing uniformly with age. It is also consistent with evidence indicating that pathways linking emotional demands, emotional labour, moral distress and burnout are largely similar across age groups, with limited age‐specific differentiation (Popucza et al. 2025; Popucza and Eriksson 2026). From this perspective, reports of disengagement among older nurses (e.g., Helaß and Maatouk 2024) might also be interpreted as reflecting cumulative exposure to emotionally demanding work together with changing life course and organizational circumstances, rather than consequences of chronological age alone.

### 4.3 Professional Identity Formation in Emotionally Demanding Work

The findings further suggest that emotional demands and emotion regulation constitute integral aspects of professional identity formation across nursing careers. Professional identity in nursing can be understood as a dynamic, experience‐based process shaped through nurses' ongoing interpretation of emotionally demanding practice situations and moral responsibilities over time (Johnson et al. 2012). Participants described how emotionally significant experiences, together with reflection on those experiences, can become integrated into their sense of professional self, values, and purpose, providing meaning, coherence, and justification that supported sustained engagement in emotionally demanding care work (Park 2010). At the same time, the findings also suggest that such identity‐based meaning‐making may not be inherently protective. Highly salient emotional experiences involving unresolved ethical conflict, lack of organizational support or perceived violations of professional integrity were described as emotionally destabilizing and, in some cases, career‐altering. This aligns with research on moral suffering and moral resilience in healthcare, which highlights how sustained moral distress may either be integrated into professional identity or contribute to its erosion, depending on available individual and organizational resources (Rushton 2023). Together, these findings suggest that professional identity formation is an ongoing process shaped by emotionally significant experiences, reflection and meaning‐making. However, whether such experiences reinforce or undermine professional identity may depend on the availability of individual and organizational resources that facilitate or hinder their integration.

### 4.4 Emotional Demands: Cumulative and Organizationally Embedded

Across participants' accounts, emotional demands were described as closely intertwined with leadership, organizational conditions, collegial relationships and other psychosocial working conditions, rather than arising solely from patient‐related interaction. While occupational health research often differentiates emotional demands from physical or cognitive demands, and from other psychosocial work factors such as workload, work pace, predictability and leadership quality (Bakker and Demerouti 2007; Berthelsen et al. 2020), nurses' narratives suggest that these dimensions are experienced as intertwined in everyday work. High workload, sustained time pressure and organizational constraints were described as emotionally taxing, giving rise to persistent anxiety, frustration, moral distress and feelings of inadequacy when nurses were unable to provide care aligned with professional expectations and values. In this sense, psychosocial working conditions themselves emerged as important sources of emotional burden through continuous exposure to emotionally demanding organizational conditions.

This interpretation aligns with research showing that psychosocial work factors are strongly associated with burnout and reduced mental well‐being in healthcare and care work (Dall'Ora et al. 2020). The present findings add qualitative depth by showing how analytically distinct work demands and psychosocial factors are experienced in practice as a continuous source of emotional exposure. Together, these findings add qualitative insight into why organizational conditions are consistently associated with burnout and reduced well‐being in nursing by illustrating how emotional demands are embedded within everyday organizational and psychosocial working conditions rather than arising solely from patient care.

### 4.5 Societal Transformations and the Intensification of Emotional Demands

The findings further indicate that participants' descriptions of changing emotional demands can be understood in relation to broader healthcare transformations, consistent with international and Swedish research describing systems increasingly characterized by efficiency pressures and administrative control (Hasselbladh et al. 2008; Mahmoud et al. 2021). From this perspective, participants' accounts suggest that emotional demands in nursing may be intensified and reshaped as care is delivered under changing structural and organizational conditions.

Participants' accounts also provide insight into broader changes in healthcare practice, workforce composition and patient care that were described as reshaping the emotional landscape of nursing work. Participants described changes in workforce stability, perceived professional competence, and communication, altering the emotional demands associated with collaboration, mentoring, and maintaining professional standards. Changes in patient characteristics and expectations, increasing clinical complexity, and advances in medical and digital technologies were described as altering both the nature of care and the emotional engagement required of nurses. Participants further described changing norms regarding emotional expression at work, including heightened expectations of emotional availability alongside shifting opportunities for emotional openness and processing. Taken together, participants' accounts suggest that changes in emotional demands and emotion regulation across nursing careers occur alongside broader transformations in healthcare systems, workforce conditions, and patient care, reflecting changing care and professional expectations rather than age‐related change alone.

### 4.6 Implications

The findings have implications for how age‐related differences in emotional demands, emotional labour, and burnout are interpreted in nursing research and practice. Rather than supporting an interpretation of linear age‐related gains or losses, participants' accounts suggest that emotional demands and their outcomes reflect cumulative, contextual processes, unfolding across long nursing careers. From this perspective, age‐related variations described by participants may be better understood in relation to accumulated professional exposure, changing life circumstances, and organizational conditions than as a direct consequence of chronological age or motivational shifts alone. These findings suggest that emotion regulation may be better understood within the realities of nursing work and across the working lifespan, rather than being attributed primarily to aging itself. Accordingly, future longitudinal research across career stages could further clarify how professional and personal dimensions, workplace features, and societal transformations shape emotional demands and emotion regulation across the working lifespan, including the relative influence of chronological age and professional experience.

The findings indicate that leadership and managerial practices were described as important in shaping how emotional demands are experienced and managed in nursing work. Leadership approaches that acknowledge emotional strain, facilitate opportunities for dialogue, and provide follow‐up after emotionally demanding events may better support nurses' opportunities for emotional processing. Conversely, participants' accounts suggest that treating emotional strain primarily as an individual responsibility rather than as an inherent aspect of nursing work may contribute to greater emotional burden. These findings suggest that managerial practices that normalize emotional demands, facilitate dialogue, and ensure ethical and emotional follow‐up may help create organizational conditions that support the management of emotionally demanding work.

The findings further underscore the importance of organizational structures that support emotional processing as part of everyday work. Opportunities for reflection, debriefing, and collegial support were often described as informal, inconsistent or absent, leaving emotional coping largely to individual nurses. At the same time, high workload, staffing shortages, and limited opportunities for rest and recovery were described as intensifying emotional strain and restricting nurses' capacity to process emotionally demanding experiences. Together, these findings suggest that organizational approaches that balance expectations, workload and staffing levels while ensuring time and space for recovery and emotional processing may better support nurses in managing emotionally demanding work.

Finally, the findings suggest the importance of work design that accommodates changing work roles, recovery needs, professional priorities and personal resources across the working lifespan. While late‐career nurses described increased confidence, they also reported cumulative fatigue, heightened sensitivity to organizational stressors and changing recovery needs. Work environments that do not accommodate these changing needs may make it more difficult for experienced nurses to sustain engagement in patient care. Adjustments to workload, work pace and role expectations, as well as opportunities to utilize experienced nurses' expertise through mentoring and supervision, may be important for supporting sustained participation in working life. Creating roles that enable experienced nurses to contribute through mentoring, supervision, and professional guidance may provide one way of drawing on accumulated expertise while also accommodating changing needs across long nursing careers.

### 4.7 Methodological Considerations

By focusing on late‐career nurses aged 60 years and older with at least 20 years of professional nursing experience, this study provides a unique qualitative perspective on emotional demands and emotion regulation across long nursing careers. The use of qualitative content analysis enabled a systematic and data‐near exploration of participants' accounts, supporting transparency and credibility. Including nurses from diverse care settings broadens the range of experiences represented and may support readers' assessment of the relevance of the findings to other contexts. First, although retrospective reflection enabled participants to describe long‐term changes and continuities across extended careers, such accounts should be understood as current reconstructions of earlier experiences rather than as objective historical records. Retrospective interviewing may involve recall limitations. Qualitative life‐course interviewing can nevertheless enable participants to reflect on and reconstruct earlier experiences over time (Parry et al. 1999). Second, because the study included only nurses aged 60 years and older reflecting on long nursing careers, it was not possible to distinguish the influence of chronological age from accumulated professional experience, career stage, changing professional roles and responsibilities, cohort effects or broader changes in healthcare over time. Comparative, quantitative, and longitudinal studies including nurses across career stages are needed to further examine these relationships. Third, the study was conducted within the Swedish healthcare context, which may limit the transferability to other countries and cultures, although similar organizational and societal challenges have been reported internationally. Fourth, recruitment through Facebook and voluntary participation may have attracted nurses with a particular interest in the topic or willingness to share their experiences. Fifth, focusing on nurses who remained in patient care later in their careers may underrepresent the experiences of those who left patient care or changed careers due to emotional strain. Consequently, the findings primarily reflect the experiences of nurses who remained in clinical practice beyond the age of 60 and may not fully represent those who exited earlier, reflecting potential selection bias, including survivorship or healthy worker effects. Sixth, some questions included specific descriptions or examples of emotional demands, emotional labour and emotion regulation and may therefore have influenced participants' responses. However, clarification of these phenomena was necessary to ensure that participants understood what was being asked. Finally, the predominance of women in the sample reflects the gender composition of the nursing workforce but limits the representation of male nurses' experiences. Experiences of emotional demands and emotion regulation may also vary according to gender‐related factors that could not be explored within the present sample.

## 5 Conclusion

Participants' accounts suggest that changes in emotional demands and emotion regulation across long nursing careers cannot be understood solely in relation to chronological age. Late‐career nurses describe changes in emotional demands and emotion regulation in relation to the development of professional emotional competence, professional identity formation and changing life course circumstances, while also illustrating how workplace features and broader societal transformations shape emotional demands and opportunities for emotion regulation throughout nursing careers. By foregrounding late‐career nurses' perspectives, the findings challenge simplified assumptions that emotional demands and emotion regulation can be understood solely in relation to chronological age. The findings also suggest that supporting emotional sustainability in nursing may warrant organizational approaches that recognize emotional demands as inherent, cumulative and embedded within organizational structures rather than viewing them primarily as patient‐related or individual responsibilities.

## Author Contributions

**Tímea Zsuzsanna Popucza:** conceptualization, methodology, data curation, project administration, writing – review and editing, writing – original draft, formal analysis, investigation, visualization. **Bernice Skytt:** conceptualization, methodology, formal analysis, supervision, writing – review and editing, validation.

## Funding

This study was supported by internal funding from the University of Gävle. The funder had no role in the study design; data collection, analysis, or interpretation; or in the writing of the manuscript.

## Disclosure

Reference count justification: This manuscript includes more than 25 references to ensure appropriate coverage of (a) methodological literature underpinning qualitative content analysis and qualitative reporting standards, and (b) the breadth of empirical and theoretical literature necessary to contextualize the study's findings and discussion. The reference list has been curated to include primarily recent, peer‐reviewed sources relevant to nursing and emotion research.

## Conflicts of Interest

The authors declare no conflicts of interest.

## Supporting information


**Data S1:** nop270957‐sup‐0001‐Supinfo1.docx.

**Data S2:** This supplementary file presents the questions included in the semi‐structured interview guide, including the descriptions and examples used to explain the phenomena of interest, all of which were asked of all participants. Additional follow‐up questions were formulated flexibly during the interviews to encourage elaboration and clarification.

**Data S3:** Illustrative examples of the analytic process from condensed meaning unit to code, category, and main category.

## Data Availability

The data that support the findings of this study are available on request from the corresponding author. The data are not publicly available due to privacy or ethical restrictions.
