# Supplementary material for: Changes in Emotional Demands and Emotion Regulation as Described by Late‐Career Nurses
Source: Nurs Open. 2026 Oct 22;13(10):e70957. doi: 10.1002/nop2.70957 (PMC13691175; doi:10.1002/nop2.70957)
Supplement: Supplementary file 1 [file NOP2-13-e70957-s001.docx]

**Supplementary** **Material S3**. Illustrative examples of the analytic process from condensed meaning unit to code, category, and main category

| **Condensed and de-identified meaning unit** | **English representation of the condensed and de-identified meaning unit** | **Code** | **Category** | **Main Category** |
| --- | --- | --- | --- | --- |
| jag hade fantastiska, empatiska, lyhörda, skickliga förebilder som hade en otrolig respekt för alla patienter och de var ledstjärnor och idoler för mig. Sen fanns det avskräckande exempel jag ville aldrig bli. | I had fantastic, empathetic, attentive, and highly skilled role models who had enormous respect for every patient. They were guiding stars and idols to me. Then there were also deterrent examples of the kind of nurse I never wanted to become. | Had highly empathetic and respectful role models to look up to, alongside negative examples I was determined not to emulate | Professional identity formation | Professional and personal dimensions |
| min nuvarande chef är sjuksköterska och hon har mycket kunskap och etiskt tänk så det är mycket lättare att ha en dialog med henne som med en chef som är ekonom. | My current boss is a nurse and she has a lot of knowledge and ethical thinking, so it's much easier to have a dialogue with her than with a boss who is an economist. | Having a nurse manager with ethical awareness in current workplace makes dialogue easier compared to economy leaders | Managerial practices and leadership climate | Workplace features |
| vi har patientens perspektiv nu, man beaktar deras önskemål. det är skillnad. Vi har större ansvar nu, man blir mer känslomässigt involverad eftersom patientens vilja ska tas tillvara på. | We have the patient's perspective now, we take their wishes into account. That's a difference. We have greater responsibility now, we become more emotionally involved because the patient's wishes must be taken into account. | Greater focus on patient perspectives and wishes today increases responsibility and leads to more emotional involvement | Medical and patient-related developments | Societal transformations |

*Note.* One illustrative example is provided for each main category to demonstrate the analytic progression from condensed meaning unit to code, category, and main category. Meaning units were identified and condensed in Swedish, after which English-language codes were generated from the condensed Swedish meaning units. Examples were selected to illustrate the analytic process and were minimally edited where necessary to protect participant confidentiality.
