# Supplementary material for: Changes in Emotional Demands and Emotion Regulation as Described by Late‐Career Nurses
Source: Nurs Open. 2026 Oct 22;13(10):e70957. doi: 10.1002/nop2.70957 (PMC13691175; doi:10.1002/nop2.70957)
Supplement: Supplementary file 2 [file NOP2-13-e70957-s002.docx]

## Supplementary Material S2. This supplementary file presents the questions included in the semi-structured interview guide, including the descriptions and examples used to explain the phenomena of interest, all of which were asked of all participants. Additional follow-up questions were formulated flexibly during the interviews to encourage elaboration and clarification.

### Emotional demands

When we talk about emotional demands at work, we mean experiences that feel emotionally challenging in your work. These may be situations where emotions, your own or those of others, play a role in your work. We are primarily interested in how you yourself describe these experiences. Can you think of situations that you experience as emotionally challenging? What kinds of experiences come to mind?

### Emotional labour

When you encounter such emotional demands, that is, emotionally challenging experiences, how do you maintain your professional role? Do you hide or modify your emotional expressions in any way, or do you express what you feel? Which emotions do you hide or modify? In what situations do you do this? Do you need to do this often? How often? Do you always do it?

Do you ever consciously try to regulate your emotions by changing how you think about the situation so that you are less emotionally affected? For example, do you remind yourself that the person is in pain or feeling anxious, and therefore not take expressions of dissatisfaction personally, helping you remain calm and maintain your professional role? In what situations do you do this? Do you need to do this often? How often? Do you always do it?

### Emotion regulation

When you encounter such emotionally challenging situations, what do you do to manage your emotions, for example, to regain your calm, let go of the emotion, or recover?

### Changes in emotional demands over time

Looking back on your nursing career, were the emotional demands you have just described also emotionally demanding earlier in your career? Were there other situations that you found emotionally demanding in the past?

Have you noticed any changes in the situations that you find emotionally challenging now compared with earlier in your career, or have they remained the same?

Can you describe whether you have noticed any changes in how emotional demands affect you now compared with earlier in your nursing career?

### Changes in emotional labour over time

Looking back on your nursing career, how did you respond to emotional demands, that is, emotionally challenging experiences? How did you maintain your professional role? Did you hide or modify your emotional expressions in any way, or did you express what you felt? Which emotions did you hide or modify? In what situations did you do this? Did you need to do this often? Did you always do it?

Looking back on your nursing career, did you consciously try to regulate your emotions by changing how you thought about situations so that you would not become emotionally affected? In what situations did you do this? Did you need to do this often? Did you always do it?

Have you noticed any changes over the course of your nursing career in how you responded to emotionally challenging experiences and maintained your professional role, or has it remained the same?

Have you noticed any changes over the course of your nursing career in how you consciously regulated your emotions by changing how you thought about situations so that you would not become emotionally affected? Or has this remained the same?

### Changes in emotion regulation over time

Looking back on your nursing career, when you previously encountered such emotionally challenging situations, what did you do to manage your emotions, for example, to regain your calm, let go of the emotion, or recover?

Have you noticed any changes in what you do to manage your emotions, for example, to regain your calm, let go of the emotion, or recover, or has it remained the same?

### Closing question

Is there anything else you would like to share about emotional demands, that is, emotionally challenging experiences?

Is there anything else you would like to share about how you express, hide, or regulate your emotions in your work?

*Note.* This supplementary file presents an English translation of the questions concerning the study topics included in the original Swedish semi-structured interview guide. Screening and eligibility-confirmation questions concerning participant characteristics and current work situation are not included. Additional follow-up questions were formulated flexibly during the interviews to encourage elaboration and clarification, for example by asking participants to recall another situation, provide an additional example, consider whether anything else came to mind, or elaborate further on their response.
